# Migrants, healthy worker effect, and mortality trends in the Gulf Cooperation Council countries

**DOI:** 10.1371/journal.pone.0179711

**Published:** 2017-06-20

**Authors:** Karima Chaabna, Sohaila Cheema, Ravinder Mamtani

**Affiliations:** Institute for Population Health, Weill Cornell Medicine-Qatar, Doha, Qatar; National Institute of Health, ITALY

## Abstract

The Gulf Cooperation Council (GCC) countries namely, Bahrain, Kuwait, Oman, Qatar, United Arab Emirates (UAE), and Saudi Arabia, have experienced unique demographic changes. The major population growth contributor in these countries is young migrants, which has led to a shift in the population age pyramid. Migrants constitute the vast proportion of GCC countries’ population reaching >80% in Qatar and UAE. Using Global Burden of Disease Study 2015 (GBD 2015) and United Nations data, for the GCC countries, we assessed the association between age-standardized mortality and population size trends with linear and polynomial regressions. In 1990–2015, all-cause age-standardized mortality was inversely proportional to national population size (*p*-values: 0.0001–0.0457). In Bahrain, Qatar, Oman, and Saudi Arabia, the highest annual decrease in mortality was observed when the annual population growth was the highest. In Qatar, all-cause age-specific mortality was inversely proportional to age-specific population size. This association was statistically significant among the 5–14 and 15–49 age groups, which have the largest population size. Cause-specific age-standardized mortality was also inversely proportional to population size. This association was statistically significant for half of the GBD 2015-defined causes of death such as “cirrhosis and other chronic liver diseases” and “HIV/AIDS and tuberculosis”. Remarkably, incoming migrants to Qatar have to be negative for HIV, hepatitis B and C, and tuberculosis. These results show that decline in mortality can be partly attributed to the increase in GCC countries’ population suggesting a healthy migrant effect that influences mortality rates. Consequently, benefits of health interventions and healthcare improvement are likely to be exaggerated in such countries hosting a substantial proportion of migrants compared with countries where migration is low. Researchers and policymakers should be cautious to not exclusively attribute decline in mortality within the GCC countries as a result of the positive effects of health interventions or healthcare improvement.

## Introduction

While basic population pyramid shapes are usually determined by birth and death rates, within the Gulf Cooperation Council (GCC) countries namely, Bahrain, Kuwait, Oman, Qatar, United Arab Emirates (UAE), and Saudi Arabia, net migration is the main factor affecting population size and age structure [1, 2]. These countries are unique because they experienced rapid population growth due to a massive migration of temporary labor [1, 2]. Hence, these countries have observed a rapid increase in population within a short period of time consisting predominantly of young and/or healthy people [3]. Between 1990 and 2015, the highest annual population growth rates were observed in the over 15 age groups (S1 and S2 Figs) [3]. The effect of migration on the population structure was that the largest segment of the population consisted of 15–49 age group (≥60% of the total population) among both males and females in the GCC countries [3]. Additionally, a substantial proportion of the GCC’s population consisted of migrants: 32%, 44%, 51% and 68% in Saudi Arabia (2013) [4], Oman (2015) [5], Bahrain (2013) [6] and in Kuwait (2012) respectively [7], and over 80% in Qatar and the UAE [8, 9]. These migrants were predominantly males causing a bulge on one side of the population pyramid [3].

GCC countries are also unique because they include five of the ten countries with the highest diabetes prevalence worldwide [10]. Additionally, in these countries, a large proportion of the population is obese. Obesity prevalence was estimated at 41% in Qatar [11], 40% in Kuwait [12], 36% in Bahrain [13], 33% in UAE [14], 30% in Oman [15], and 28% in Saudi Arabia [16]. Of note, obesity prevalence in these countries was similar or higher than in the United States, where it is estimated to be 36% [17]; and much higher than global estimate at 13% [18]. Overall, mortality has decreased in the GCC countries, particularly among children (S3 Fig) [19, 20]. This decrease in mortality is commonly attributed to socioeconomic development and healthcare improvements [21].

In the GCC countries, all-cause and cause-specific mortality age-standardized rates were estimated by the Global Burden of Disease, Injuries, and Risk Factors Study (GBD) in the framework of a collaborative effort quantifying the burden of disease worldwide [22]. We hypothesized that the massive migration of young and/or healthy people within a short span of time in the GCC countries, was one of the major causes of the generalized decline in age-standardized mortality rates. Using Global Burden of Disease Study 2015 (GBD 2015) data [19, 20], we assessed all-cause and cause-specific mortality trends according to national population size trends [3].

## Materials and methods

We utilized publically available data between 1990 and 2015 for the GCC countries namely, Bahrain, Kuwait, Oman, Qatar, Saudi Arabia, United Arab Emirates. Population data were retrieved from the World Population Prospect 2015 Revision by United Nations (UN) Population Division (https://esa.un.org/unpd/wpp/Download/Standard/Population/) [3]; and mortality data were retrieved from GBD 2015 (http://vizhub.healthdata.org/gbd-compare/) [19, 20]. We used UN data since GBD 2015 [19, 20] used this population data source [3] to estimate their mortality rates.

Causes of death included in our analyses were all causes and GBD 2015-defined death causes with the codes: A1-A7, B1-B10, and C1-C3. The cause of death “Forces of nature, war, and legal intervention” (GBD 2015 code C4) was not included in our analysis, as GCC countries have not been subjected to forces of nature, war, and legal intervention.

We computed annual population growth and annual percent change in all-cause age-standardized and age-specific rates. Using simple linear and polynomial regressions, we assessed the association between all-cause age-standardized death rates and population sizes for each of the GCC countries. In Qatar, we also assessed the association between all-cause age-specific death rates and age-specific population sizes; and the association between cause-specific age-standardized death rates and population sizes. We conducted analysis of variance (ANOVA) to identify, which order of the polynomial regression model should be selected (significance threshold at 0.05). We assessed significance of the association using the F-test. Association statistical significance threshold at 0.05 was corrected using Bonferroni method to address multiple testing problem. As we conducted 32 tests, significance threshold was at 0.0016. We produced diagnostic plots of fitted values versus residuals to assess randomness and unpredictability of the residuals. We assessed the goodness-of-fit of the models using multiple R-squared statistics. We used R-3.3.1 software [23] for our analyses.

## Results and discussion

**Fig 1:**
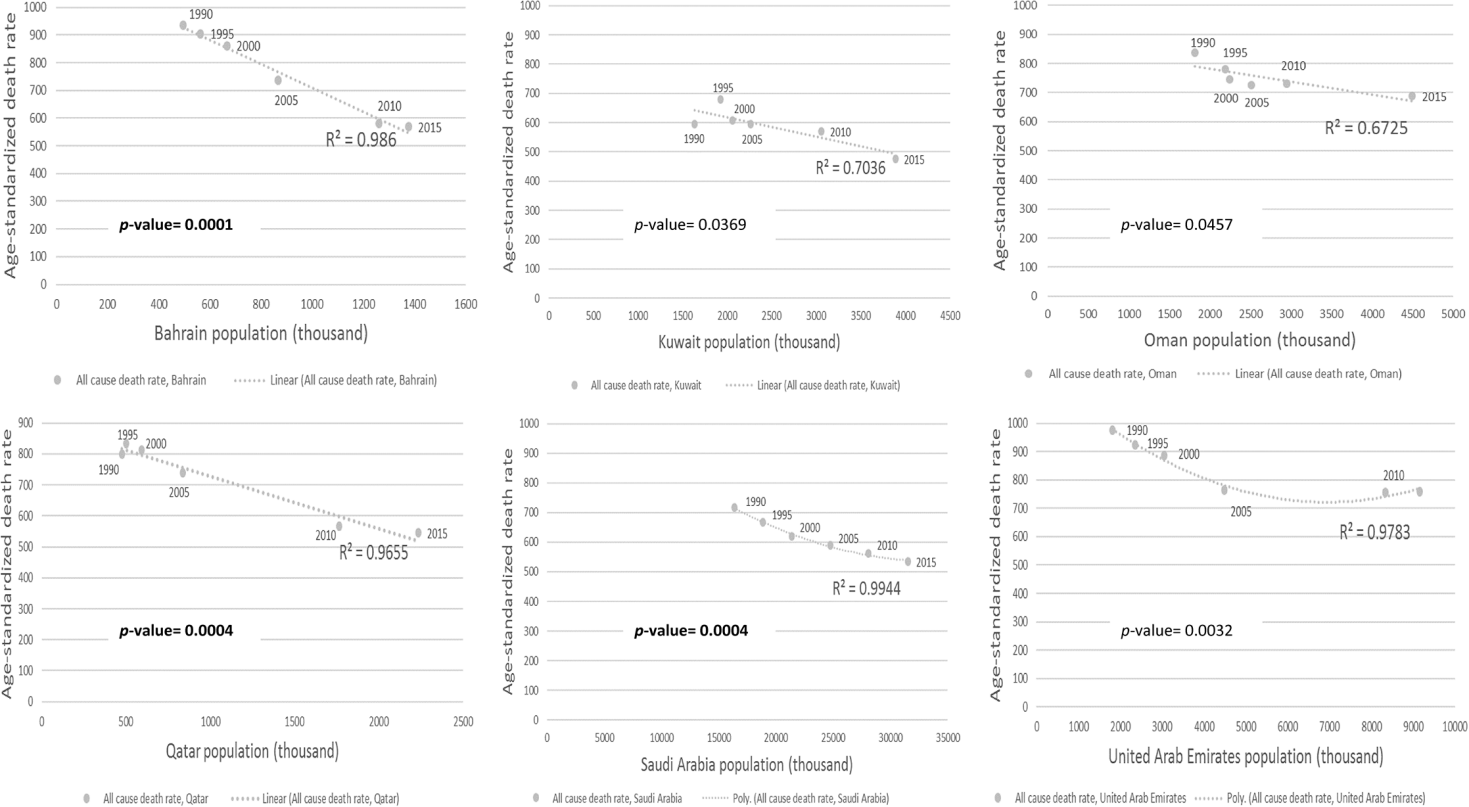
Association between all-cause age-standardized mortality and population trends in the countries of Gulf Cooperation Council (GBD 2015 and United Nation data). Triangle labels: mortality year, all-cause age-specific death rates (per 100,000), dash line: regression line.

Changes in the national population size seem to affect age-standardized rates. In the GCC countries between 1990 and 2015, all-cause age-standardized mortality rates were inversely proportional to national population size (*p*-values between 0.0001 and 0.0457) (Fig 1 and S4 Fig) [3, 19, 20]. After applying Bonferroni correction on the *p*-value, the association between all-cause age-standardized mortality and population size was statistically significant for Bahrain (*p*-value = 0.0001, R^2^ = 0.99), Qatar (*p*-value = 0.0004, R^2^ = 0.97), and Saudi Arabia (*p*-value = 0.0004, R^2^ = 0.99). This association is interesting, as the age-standardization method should remove the effect of differences in population age structures observed over time. Furthermore, this association between age-standardized mortality and population size suggests a strong healthy population effect attributed to migrants which represent a substantial proportion of the GCC countries’ population (up to >80% [8, 9]).

Migrant mortality and healthy migrant effect have been described in other regions and explained by migrant self-selection and health screening by host country, among others [24–27]. For instance in Australia, the increase in Australian life expectancy between 1981 and 2003 was attributed partly to healthy migrant effect [24]. Within the GCC countries, health screening is mandatory for migrants upon arrival. Migrants are particularly screened for infectious diseases such as HIV, hepatitis B and C, syphilis, and tuberculosis [28–33]. In the GCC countries, healthy migrant effect appears to affect country-level mortality rates. As the GCC countries’ population increases due to migration, the mortality rates further tend to decrease. Benefits of health interventions and healthcare improvements are likely to be exaggerated in such countries that host a substantial proportion of migrants compared with countries where migration is low such as Japan, Algeria, and Morocco, among others [24]. It is likely that our results are applicable to other countries with more than 30% migrant workers in their populations e.g. Andorra, Brunei, Hong Kong, Israel, Liechtenstein, Luxembourg, Singapore, and Switzerland (S5 Fig) [34].

In Qatar, until 2000, annual population growth was below 4% (Table 1) [3]. Thereafter, population growth increased rapidly reaching a peak at 22.2% between 2005 and 2010. After 2010, the population growth decreased reaching 5.3% for the period 2010–2015. Remarkably, the annual decrease in all-cause age-standardized mortality was the highest at -4.7% between 2005 and 2010. Likewise, in Bahrain, population growth was the highest in 2005–2010 at 9.1%; and during the same period, the decrease in all-cause age-standardized mortality was the highest (-4.3%). In Oman and Saudi Arabia, the highest annual decrease in mortality was also observed when annual population growth rates were the highest. However, in Kuwait and United Arab Emirates, this pattern of high decrease in mortality during high population growth was not clear.

**Table 1:** Annual percent change of all-cause mortality in the GCC countries and their annual population growth between 1995 and 2015.

Country	Year	All-cause death rate (per 100,000)	Annual percent change (%)	Population (thousand)	Annual population growth (%)
Bahrain	1990	935.1		496	
	1995	904.9	-0.6	564	2.7
	2000	861.2	-1.0	667	3.7
	2005	737.3	-2.9	867	6.0
	2010	580.1	-4.3	1,261	9.1
	2015	569.1	-0.4	1,377	1.8
Kuwait	1990	594.1		1,637	
	1995	680.3	2.9	1,929	3.6
	2000	607.0	-2.2	2,059	1.3
	2005	595.2	-0.4	2,264	2.0
	2010	570.3	-0.8	3,059	7.0
	2015	475.8	-3.3	3,892	5.4
Oman	1990	836.9		1,812	
	1995	781.4	-1.3	2,192	4.2
	2000	745.8	-0.9	2,239	0.4
	2005	725.7	-0.5	2,507	2.4
	2010	731.2	0.2	2,944	3.5
	2015	687.8	-1.2	4,491	10.5
Qatar	1990	800.6		476	
	1995	832.7	0.8	501	1.0
	2000	812.5	-0.5	593	3.7
	2005	739.5	-1.8	837	8.2
	2010	566.9	-4.7	1,766	22.2
	2015	544.7	-0.8	2,235	5.3
Saudi Arabia	1990	717.9		16,361	
	1995	666.9	-1.4	18,854	3.0
	2000	619.5	-1.4	21,392	2.7
	2005	588.8	-1.0	24,745	3.1
	2010	561.1	-0.9	28,091	2.7
	2015	535.0	-0.9	31,540	2.5
United Arab Emirates	1990	976.4		1,811	
	1995	924.1	-1.1	2,350	5.9
	2000	887.7	-0.8	3,050	6.0
	2005	764.1	-2.8	4,482	9.4
	2010	757.4	-0.2	8,329	17.2
	2015	758.9	0.0	9,157	2.0

Similarly, all-cause age-specific mortality was inversely proportional to age-specific population size in Qatar (Table 2 and S6 Fig). This association was statistically significant among the 5–14 and 15–49 age groups (*p*-value<0.001), which have the largest population size (S2 and S3 Figs). Furthermore, in all age groups, the decrease in all-cause mortality was the highest during the same 5-year period with the highest age-specific annual population growth. This systematic decline in mortality rates cannot be attributed only to improvement in healthcare since this has been observed at a gradual pace in Qatar [35]. The large increase in Qatar’s population within a short span after 2000 could partly explain the decline in mortality rates owing to a substantial increase in the denominator, while the numerator remained minimally affected. In each age group, the increase in population is observed predominantly due to the influx of young and/or healthy individuals. Hence, one of the major drivers of decreased mortality rates appears to be migration. Consequently, decline in mortality observed in Qatar’s population consisting of nationals, short-term and long-term residents should not be considered as a positive indicator of Qatar’s population health status.

**Table 2:** Simple linear and polynomial regression statistics assessing the association between all-cause age-specific mortality trends and age-specific population size trends, Qatar, both genders.

Age group	Year	All-cause death rate (per 100,000)	Annual percent change (%)	Population (thousand)	Population growth (%)	R-squared	*p*-value
Under 5 years	1990	446	-	52		0.837^a^	0.0105
	1995	397	-2.2	49	-1.2		
	2000	344	-2.7	56	2.9		
	2005	293	-2.9	72	5.8		
	2010	256	-2.5	89	4.9		
	2015	188	-5.4	132	9.7		
5–14 years	1990	40	-	83		0.988^b^	0.0013^c^
	1995	38	-1.4	84	0.2		
	2000	33	-2.4	98	3.4		
	2005	27	-3.4	125	5.4		
	2010	23	-3.6	149	3.9		
	2015	18	-4.2	215	8.8		
15–49 years	1990	159	-	306		0.953^a^	0.0008^c^
	1995	168	1.2	330	1.6		
	2000	168	-0.1	384	3.3		
	2005	138	-3.6	552	8.7		
	2010	98	-5.8	1382	30.0		
	2015	87	-2.2	1705	4.7		
50–69 years	1990	849	-	32		0.914^a^	0.0029
	1995	918	1.6	34	1.5		
	2000	909	-0.2	50	9.2		
	2005	741	-3.7	82	13.0		
	2010	498	-6.6	134	12.6		
	2015	515	0.7	167	4.9		
70+ years	1990	7,054		3		0.892^a^	0.0045
	1995	7,164	0.3	4	3.9		
	2000	6,666	-1.4	5	6.3		
	2005	6,111	-1.7	6	2.4		
	2010	3,988	-6.9	12	19.6		
	2015	4,080	0.5	17	8.1		

a: simple linear regression

b: polynomial regression order 2

c: statistically significant (F-test)

**Fig 2:**
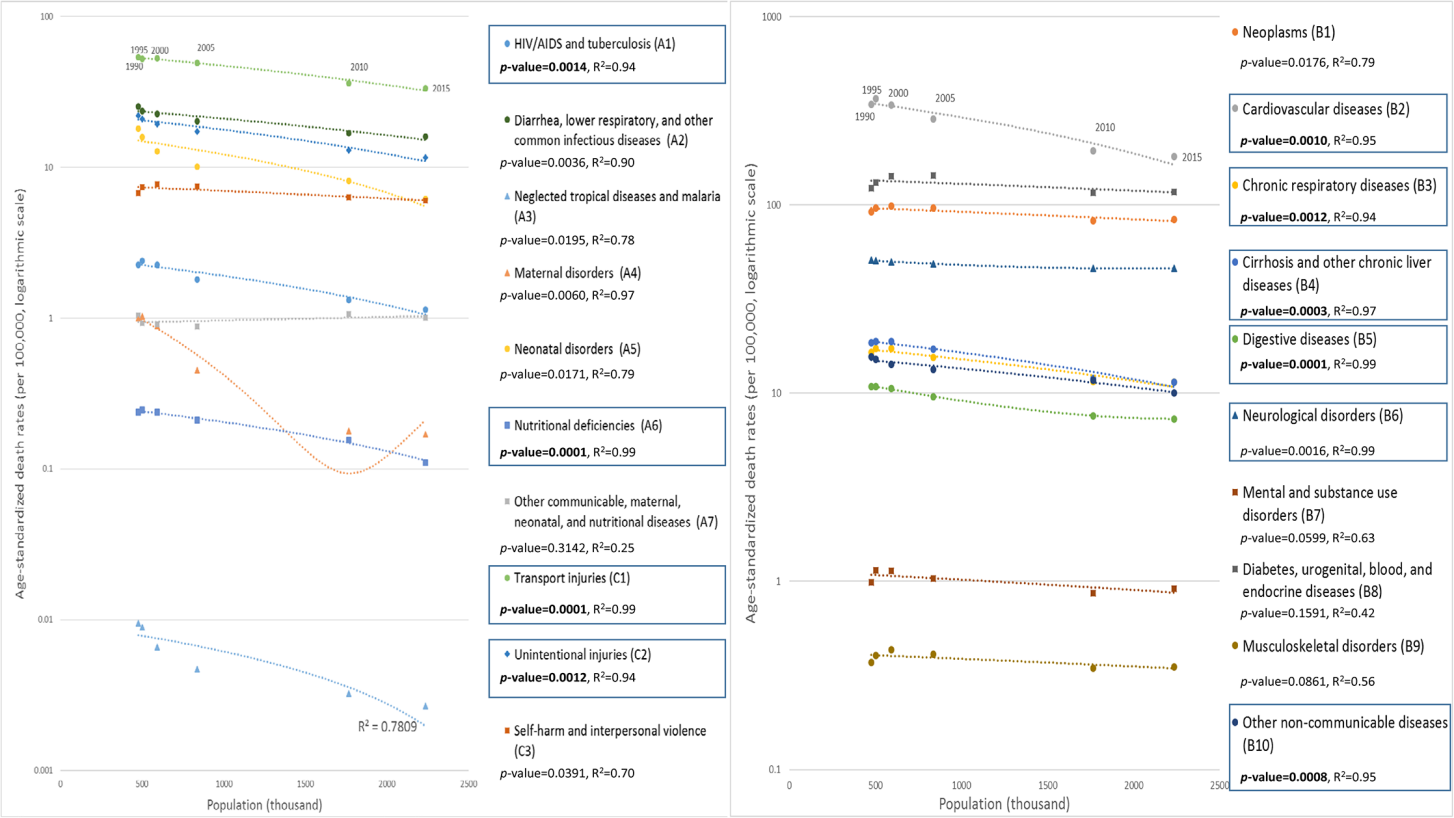
Cause-specific age-standardized mortality against population size in Qatar, both genders (GBD 2015 and United Nation data). Dash line: regression line.

In Qatar, cause-specific age-standardized mortality rates were also inversely proportional to population size (Fig 2 and S7 Fig). This association was statistically significant for half of the GBD 2015-defined causes of death such as “cirrhosis and other chronic liver diseases” (B4, *p*-value<0.001, R^2^ = 0.97) and “HIV/AIDS and tuberculosis” (A1, *p*-value<0.01, R^2^ = 0.94). Interestingly, incoming migrants to Qatar have to be negative for HIV, hepatitis B and C, and tuberculosis [30, 31], which could further explain the decrease in cause-specific mortality for “HIV/AIDS and tuberculosis” (A1) and “cirrhosis and other chronic liver diseases” (B4). Therefore, cause-specific mortality rates appear to be influenced by the health profile of incoming migrants to Qatar.

In their methodology, GBD 2015 used three updated death distribution methods to estimate completeness of death registration [19, 20]. These demographic methods were also used to estimate the fraction of deaths counted by registration systems [36]. Correction factors for observed deaths are produced by these methods to estimate mortality levels [36]. Remarkably, these three methods should be applicable only if there is no migration observed in the studied population [36]. We can argue that these death distribution methods cannot be applied to the GCC countries, since the major contributor of population growth is migrant labor [1, 2]. Hence, mortality rates for the GCC countries estimated by GBD 2015 should be interpreted with caution [36]. In addition, as years of life lost and disability-adjusted life year are estimated from mortality, these metrics used to quantify the burden of disease within the GCC countries should also be interpreted with caution.

In our study, we used publically available GBD 2015 and UN data, to assess the association between mortality and population trends in the GCC countries. The strength of our study is that we emphasize demographic specificities in the GCC countries that should be taken into consideration when interpreting mortality rates in these countries. This has relevance and significance for developing and monitoring population health programs. Policy makers may use the mortality rates as available in the GBD data without distinguishing between nationals, long-term residents and migrants. Our analysis was limited as GBD 2015 and UN data do not provide data by sub-populations (migrants *versus* Qataris and long-term residents). As such, we were not able to compare mortality in the total population with mortality in the subpopulations to further demonstrate our hypothesis of healthy migrant effect.

In conclusion, our findings show that the increase in GCC countries’ population over the last two decades have decreased mortality rates. This increase in population is due to an influx of large number of migrants, which constitute a substantial proportion of GCC countries’ population. Mortality trends might have been varied if the current migration strategy in the GCC countries were different. In general, researchers and policymakers in the GCC countries should be cautious so as not to exclusively attribute this decline in mortality rates as a result of the positive effect of health-based interventions or improvement in the healthcare system. Furthermore, mortality decline observed in the total population (nationals and short-term and long-term residents) of the GCC countries should not be considered as a positive indicator for population health status. In order, to elucidate changes in mortality trends as a result of health-based interventions and improvement in the healthcare system, stratification—nationals and long-term residents (≥15 years) *versus* short-term residents (<15 years), should be considered [37].

## Supporting information

S1 Fig Population size time trends in the countries of Gulf Cooperation Council among males by age group (United Nation data). (TIF)

S2 Fig Population size time trends in the countries of Gulf Cooperation Council among females by age group (United Nation data). (TIF)

S3 Fig Steady decline in all-cause age-specific mortality rates among children and in cause-specific age-standardized mortality rates for neonatal disorders, in the countries of the Gulf Cooperation Council, males and females (GBD 2015 data). (TIF)

S4 Fig Diagnostic plots–fitted values versus residuals, of the linear and polynomial regression assessing the association between all-cause mortality and population trends in the countries of Gulf Cooperation Council. (TIF)

S5 Fig Plots of population size versus all-cause age-standardized mortality in Andorra, Brunei, Israel, Luxembourg, Singapore, and Switzerland (GBD 2015 and United Nation data). (TIF)

S6 Fig Diagnostic plots–fitted values versus residuals, of the linear and polynomial regression assessing the association between all-cause age-specific mortality trends and age-specific population size trends, Qatar, both genders. (TIF)

S7 Fig Diagnostic plots–fitted values versus residuals, of the linear and polynomial regression assessing the association between cause-specific age-standardized mortality against population size, Qatar, both genders. (TIF)

## Abbreviations

GBD: Global Burden of Disease, Injuries, and Risk Factors Study
GBD 2015: Global Burden of Disease Study 2015
GCC: Gulf Cooperation Council
UAE: United Arab Emirates
S: Supplementary information
