# Supplementary figures and images for: Migrants, healthy worker effect, and mortality trends in the Gulf Cooperation Council countries
Source: PLoS One. 2017 Jun 20;12(6):e0179711. doi: 10.1371/journal.pone.0179711 (PMC5478152; doi:10.1371/journal.pone.0179711)

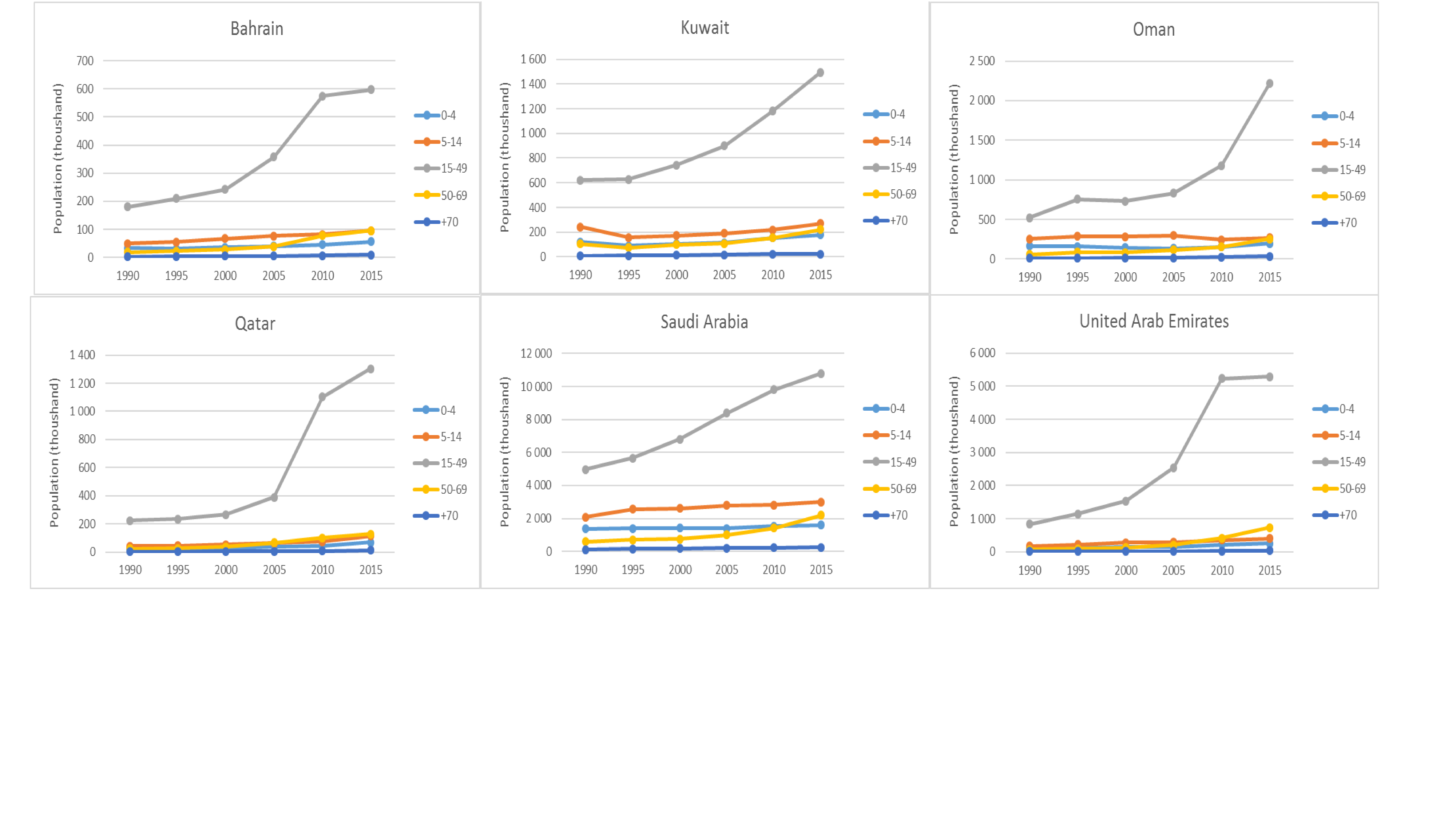

Supplement: S1 Fig — (TIF) [file pone.0179711.s001.tif]

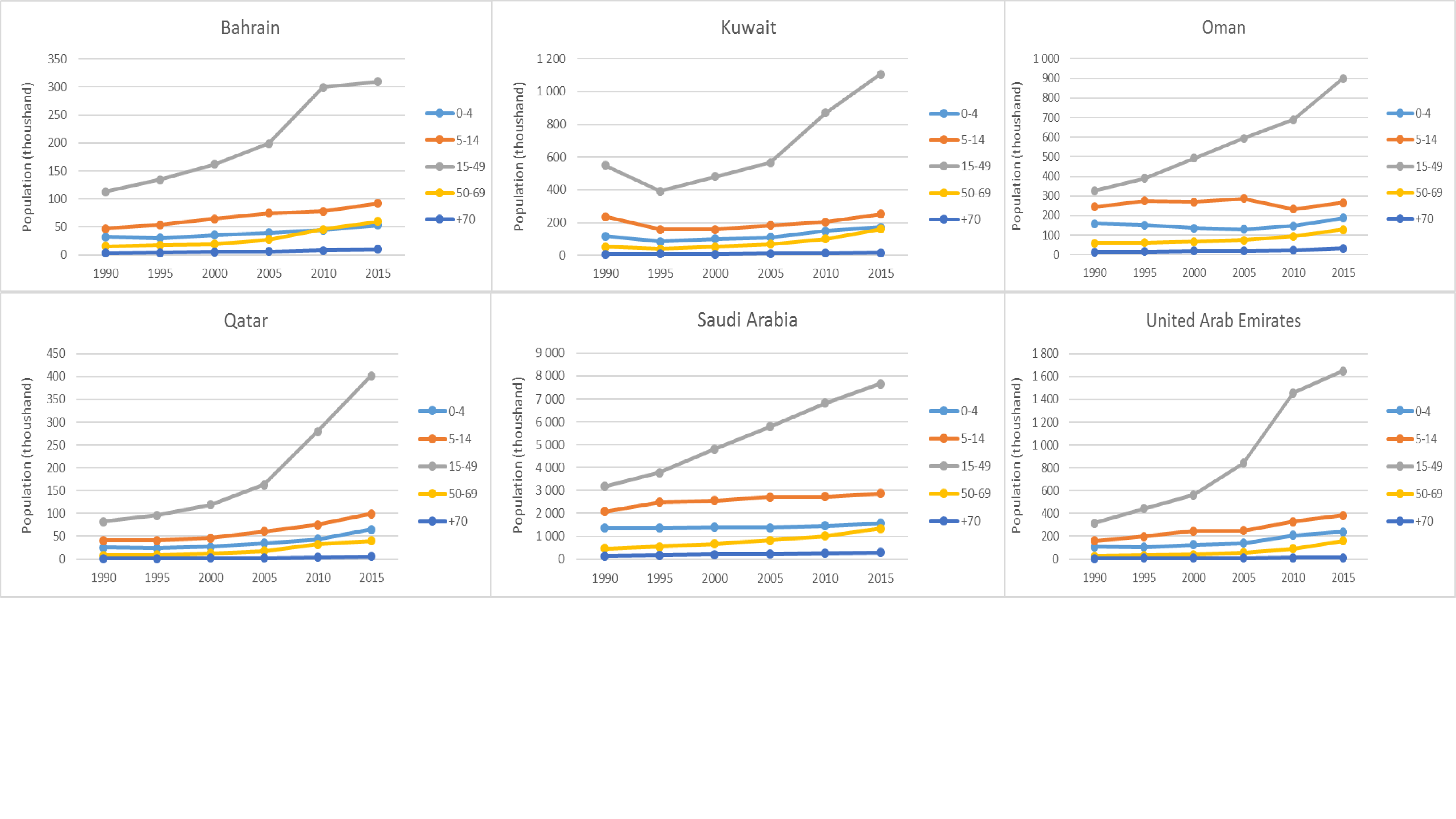

Supplement: S2 Fig — (TIF) [file pone.0179711.s002.tif]

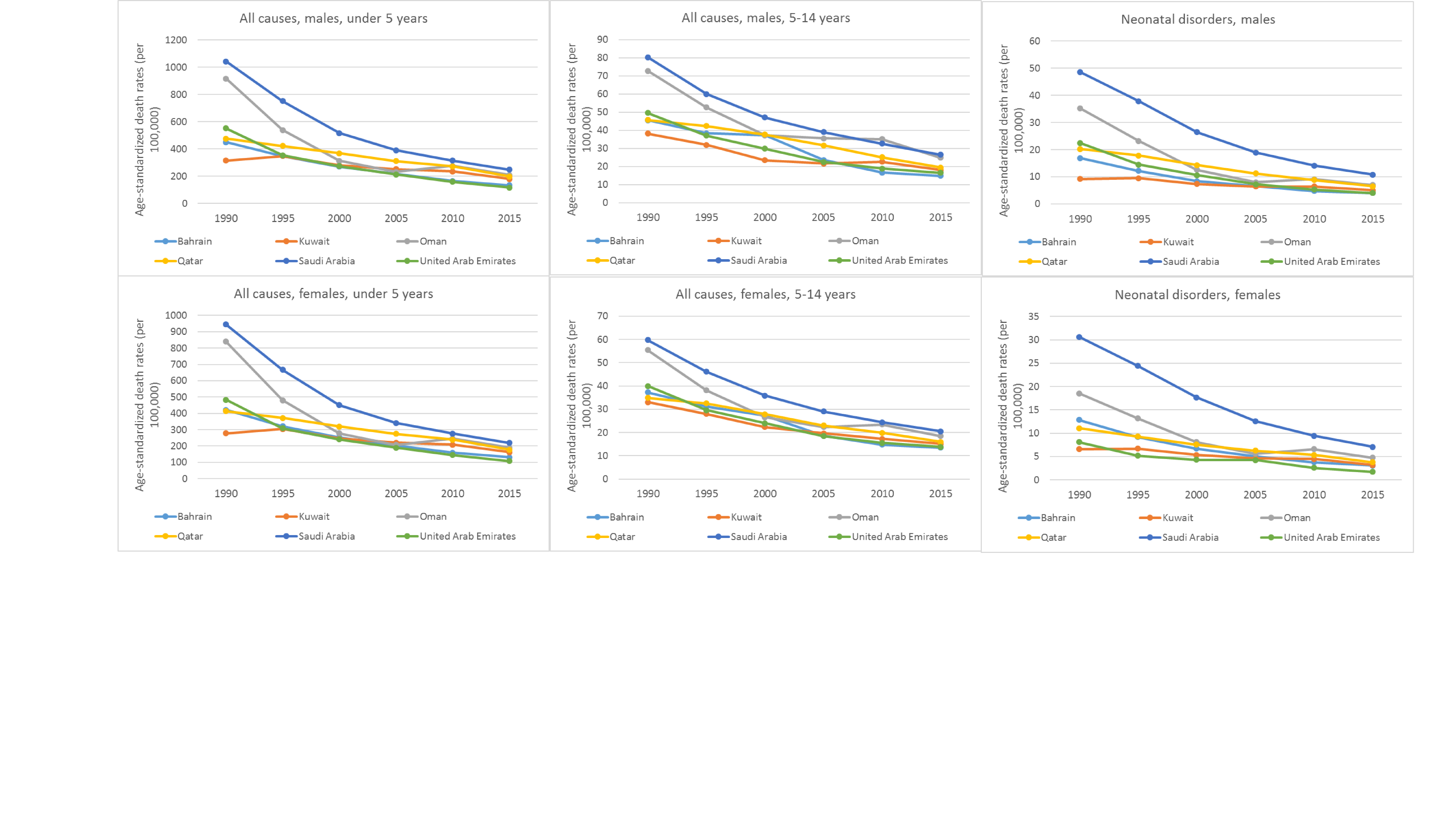

Supplement: S3 Fig — (TIF) [file pone.0179711.s003.tif]

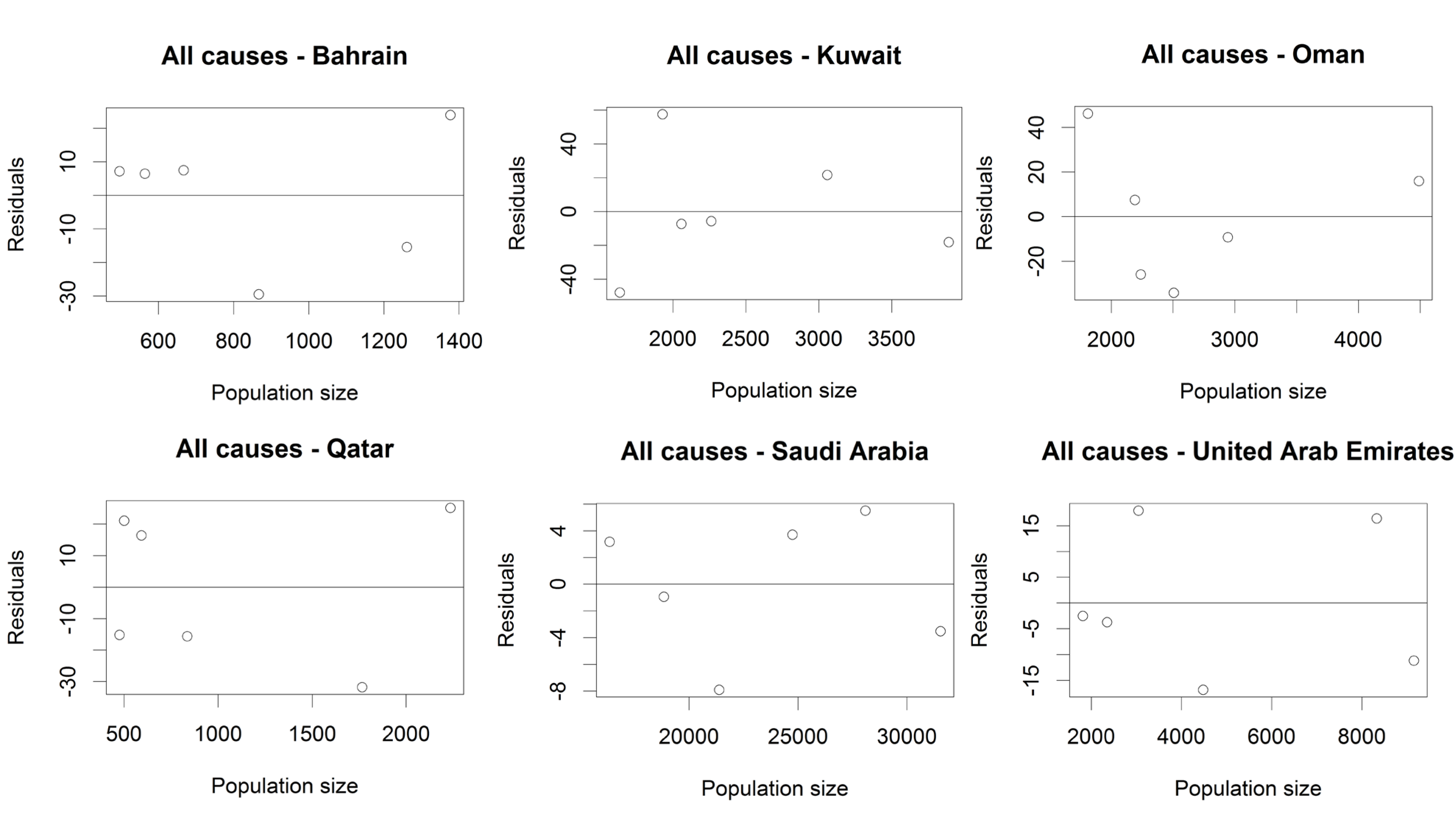

Supplement: S4 Fig — (TIF) [file pone.0179711.s004.tif]

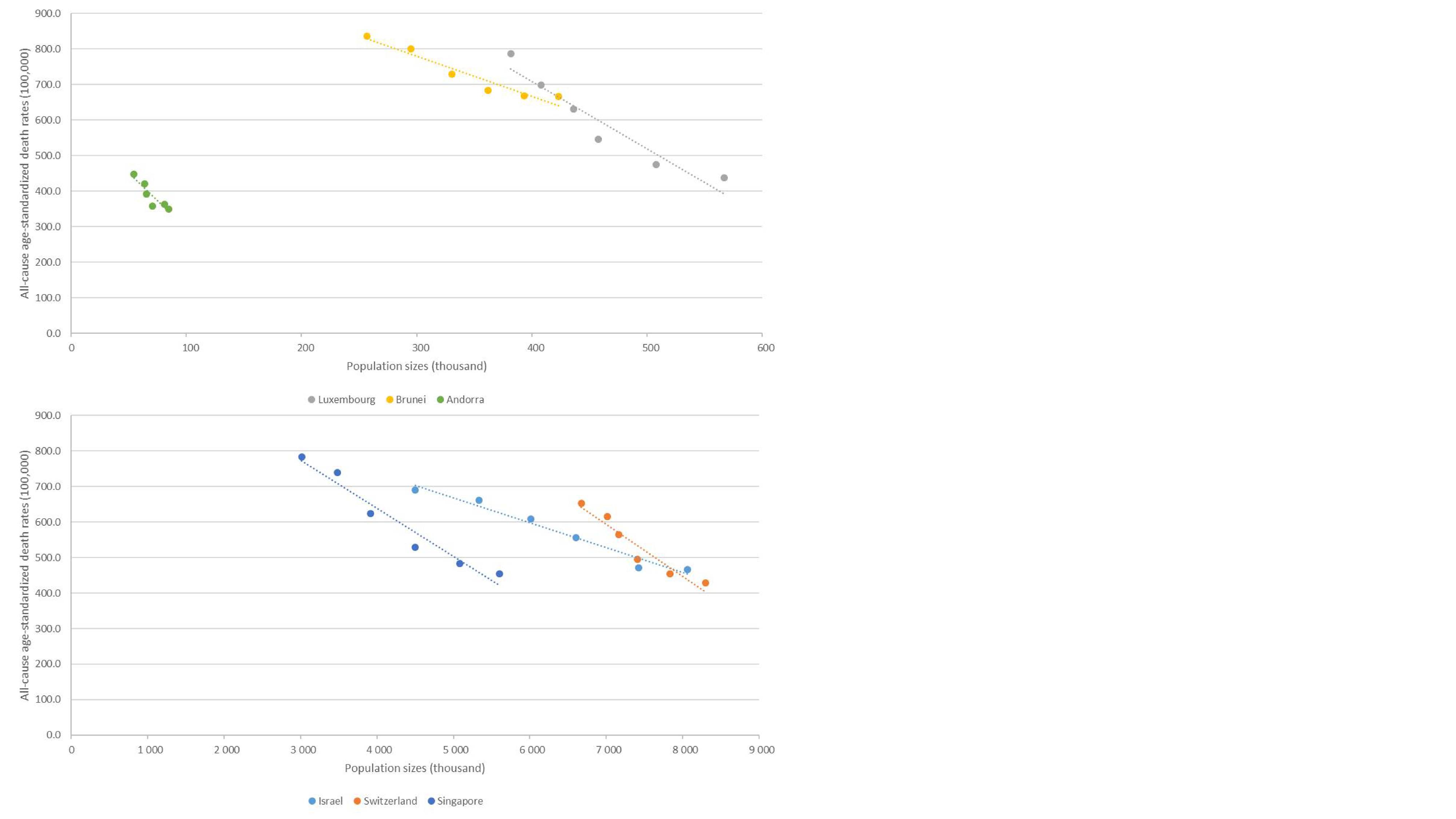

Supplement: S5 Fig — (TIF) [file pone.0179711.s005.tif]

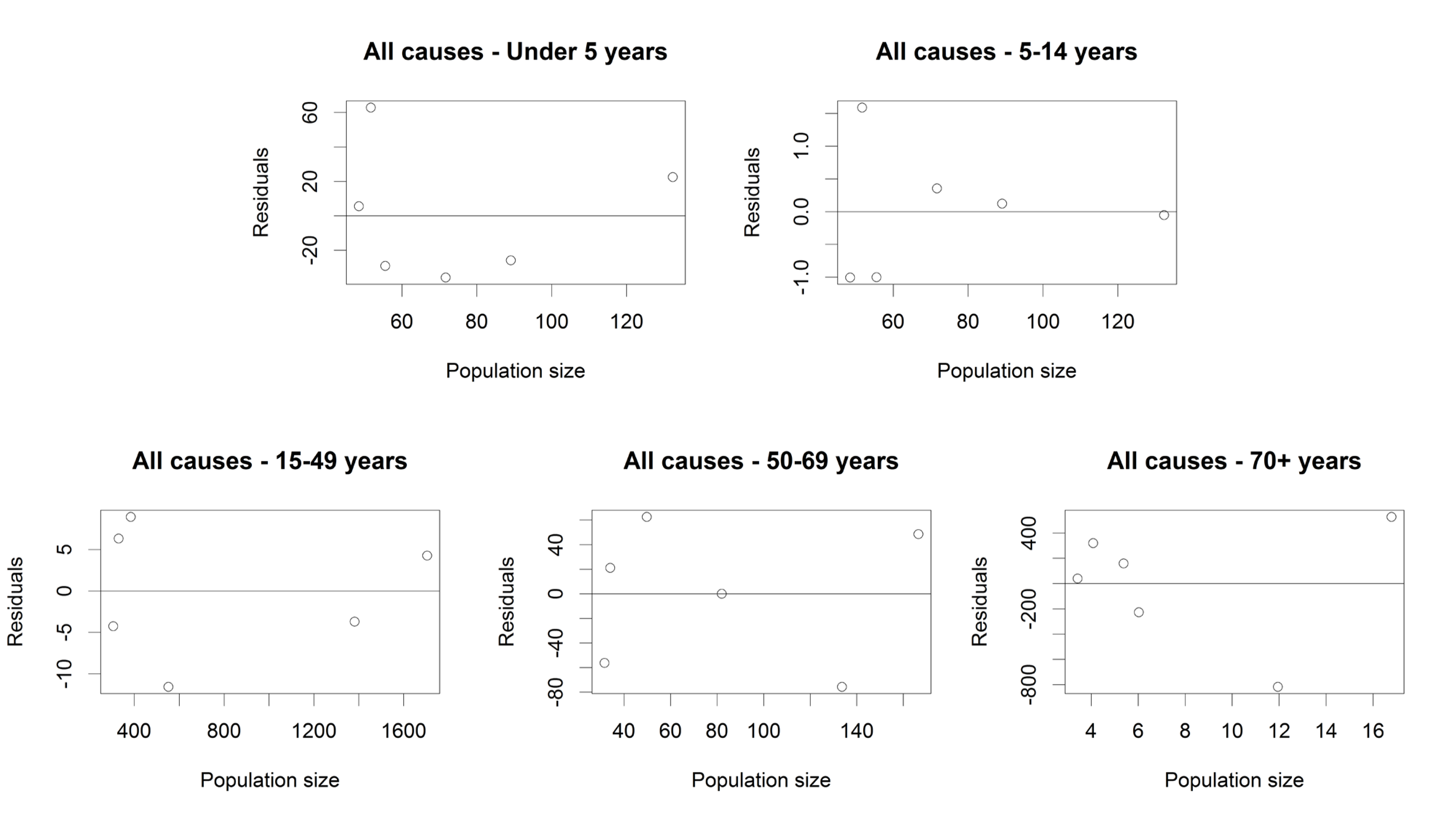

Supplement: S6 Fig — (TIF) [file pone.0179711.s006.tif]

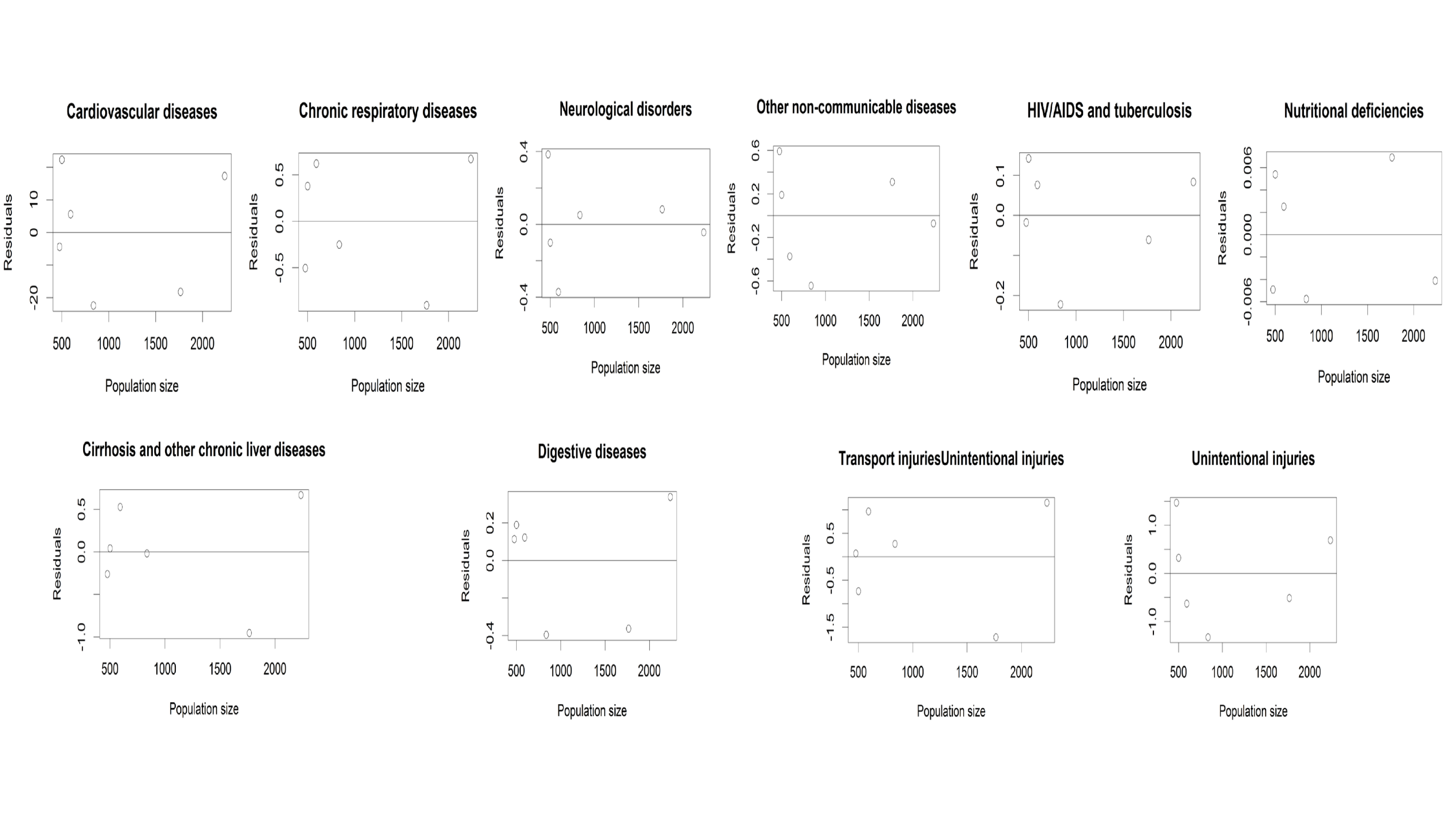

Supplement: S7 Fig — (TIF) [file pone.0179711.s007.tif]
